# Supplementary material for: Changes in Gut Microbiota Induced by Doxycycline Influence in Vascular Function and Development of Hypertension in DOCA-Salt Rats
Source: Nutrients. 2021 Aug 26;13(9):2971. doi: 10.3390/nu13092971 (PMC8464928; doi:10.3390/nu13092971)
Supplement: Supplementary file 1 [file nutrients-13-02971-s001.zip › nutrients-1335415-SI.pdf]

Supplementary Materials:

Table S1. Oligonucleotides for real-time RT-PCR.

mRNA Targets	Descriptions	Sense	Antisense
TNF-α	Tumor necrosis factor-alpha	ACGATGCTCAGAAACACACG	CAGTCTGGGAAGCTCTGAGG
IL-1β	Interleukin-1beta	GTCACTCATTGTGGCTGTGG	GCAGTGCAGCTGTCTAATGG
IL-6	Interleukin-6	GATGGATGCTTCCAAACTGG	AGGAGAGCATTGGAAGTTGG
NOX-1	NOX-1 subunit of NADPH oxidase	TCTTGCTGGTTGACACTTGC	TATGGGAGTGGGAATCTTGG
NOX-2	NOX-2 subunit of NADPH oxidase	ATGCAGGAAAGGAACAATGC	TTGCAATGGTCTTGAACTCG
p22phox	p22phox subunit of NADPH oxidase	GCGGTGTGGACAGAAGTACC	CTTGGGTTTAGGCTCAATGG
p47phox	p47phox subunit of NADPH oxidase	ATGACAGCCAGGTGAAGAAGC	CGATAGGTCTGAAGGCTGATGG
FoxP3	Forkhead box P3	AGGCACTTCTCCAGGACAGA	CTGGACACCCATTCCAGACT
IL-10	Interleukin-10	CCAGCTGGACAACATACTGC	AGGGTCTTCAGCTTCTCACC
RORγ	ROR-gamma	GCCTACAATGCCAACAACCACACA	TGATGAGAACCAAGGCCGTGTAGA
IL-17a	Interleukin-17a	TCAGACTACCTCAACCGTTCC	CAGTTTCCCTCCGCATT
TLR-4	Toll-like receptor-4	GCCTTTCAGGGAATTAAGCTCC	AGATCAACCGATGGACGTGTAA
IAP	Intestinal alkaline phosphatase	CAT GGA CCG CTT CCC ATA	CTT GCA CTG TCT GGA ACC TG
IL-18	Interleukin-18	GACTCTTGCGTCAACTTCAAGG	CAGGCTGTCTTTTGTCAACGA
Occludin	Occludin	ACGGACCCTGACCACTATGA	TGGAGATGAGGCTTCTGCTT
Muc-2	Mucin-2	GATAGGTGGCAGACAGGAGA	GCTGACGAGTGGTTGGTGAATG
Muc-3	Mucin-3	CACAAAGGCAAGAGTCCAGA	AGTGTCCTTGGTGCTGCTGAATG
ZO-1	Zonula occludens-1	GGGGCCTACACTGATCAAGA	TGGAGATGAGGCTTCTGCTT
RPL13	Ribosomal protein L13	CCTGCTGCTCTCAAGGTTGTT	TGGTTGTCACTGCCTGGTACTT
GAPDH	Glyceraldehyde-3-phosphate dehydrogenase	TGCACCACCAACTGCTTAGC	GGATGCAGGGATGATGTTCT

Table S2. Morphological parameters of all experimental groups.

Variables	Ctrl (n = 8)	DOCA (n = 8)	DOCA+DOX (n = 8)
**BW (g)**	352.4 ± 18.7	347.5±11.1**	350.3 ± 13.2
**HW (mg)**	927.8 ± 31.1	1267.4 ± 50.8**	1091.6 ± 41.8#
**LVW (mg)**	637.1 ± 23.1	905.1 ± 43.6**	783.28 ± 36.7#
**KW (mg)**	1037.4 ± 41.7	2253.2 ± 155.1**	2148.2 ± 130.2
**HW/TL (mg/cm)**	176.8 ± 4.9	238.7 ± 7.0**	209.2 ± 7.9#
**LVW/TL (mg/cm)**	121.4 ± 3.6	170.4 ± 6.3**	150.12 ± 6.8#
**KW/ TL (mg/cm)**	197.6 ± 6.5	423.7 ± 24.8**	413.2 ± 25.1

BW, Body weight; HW, Heart weight; KW, Kidney weight; LVW, Left ventricular weight; TL, Tibia length. Results are shown as mean ± SEM. All parameters were assessed in DOCA-salt rats treated with vehicle or Doxycycline (DOX). **P < 0.01 *vs.* control (Ctrl) group; #P < 0.05 and ##P < 0.01 *vs.* DOCA-salt group.

Table S3. Effects of Doxyciclyne (DOX) treatment on phyla changes in the gut microbiota.

Phylum	Ctrl (n = 6)	DOCA (n = 6)	DOCA+Dox (n = 6)
**Firmicutes**	72.65± 4.81	78.65 ± 3.49	75.38 ± 4.78
**Bacteroidetes**	24.96 ± 4.42	18.98 ± 3.38	23.89 ± 4.80
**Tenericutes**	1.50 ± 0.71	0.75 ± 0.43	0.00 ± 0.00
**Proteobacteria**	0.44 ± 0.30	0.74 ± 0.34	0.10 ± 0.04
**Cyanobacteria**	0.10 ± 0.03	0.02 ± 0.01*	0.17 ± 0.08
**Actinobacteria**	0.07 ± 0.02	0 .41± 0.15*	0.07 ± 0.04#
**Others**	2.76 ± 0.12	0.24 ± 0.01	0.15 ± 0.01

Values are expressed as mean ± SEM (n = 6). *P< 0.05 compared with the Ctrl group. #P< 0.05 compared with the DOCA-salt group.

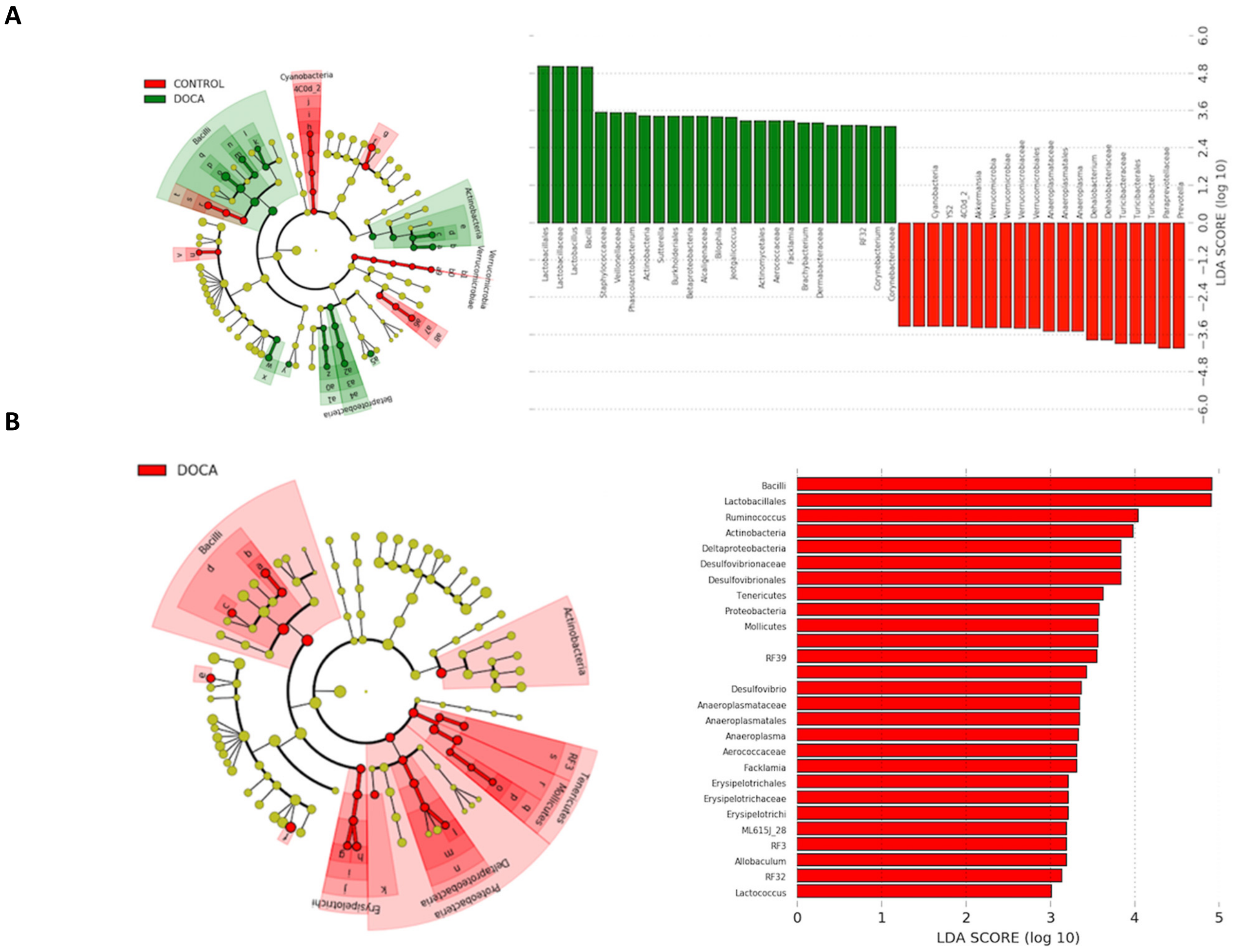

Figure S1. *Changes in the gut microbiota composition in the DOCA-salt model of hypertension.* Comparisons of microbiome changes in control *versus* Deoxycorticosterone Acetate (DOCA)-salt hypertensive rats (**A**). Linear discriminant analysis effect size (LEfSe) identified significantly different bacterial taxa enriched in each cohort at LDA Score > 2, $p < 0.05$ (red bars CONTROL enriched, green bars DOCA enriched). Microbiome changes in DOCA-salt rats *versus* DOCA-salt treated with doxycycline (**B**). Linear discriminant analysis effect size (LEfSe) identified significantly different bacterial genera representing > 0.1% of total bacteria enriched in each cohort at LDA Score > 2, $p < 0.05$ (red bars DOCA enriched). Cladograms show the significantly enriched taxa, the taxa are identified in the key of each panel. Larger circles represent greater differences in abundance between groups (**A**,**B**). n = 6 animals per treatment group for each comparison.

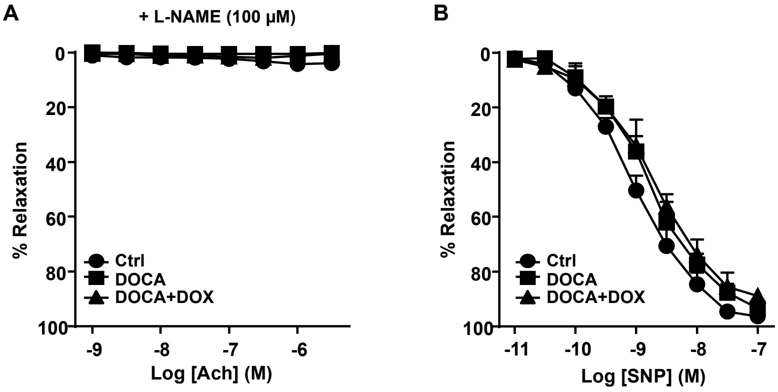

Figure S2. Effects of doxycycline (DOX) treatment on vascular nitric oxide pathway. Vascular relaxation responses induced by acetylcholine (Ach) in endothelium-intact aortae pre-contracted by phenylephrine (1 μM) in the absence and in the presence of N^G-nitro-L-arginine methyl (L-NAME) (**A**). Endothelium-independent vasodilator responses to sodium nitroprusside (SNP) (**B**) in endothelium-denuded aortae pre-contracted by phenylephrine (1 μM) in all experimental groups. Values are expressed as mean ± SEM (n = 6-8).

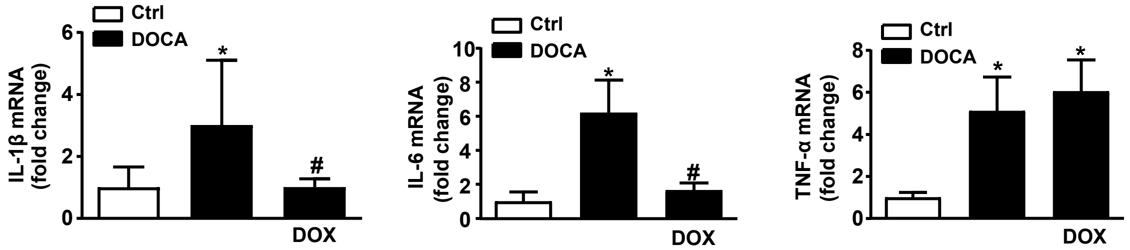

Figure S3. Effects of doxycycline (DOX) treatment on vascular inflammation. Aortic mRNA levels of pro-inflammatory cytokines measured by RT-PCR in control (Ctrl) and DOCA-salt rats. Values are expressed as mean ± SEM (n = 8). * $p < 0.05$ and ** $p < 0.01$ compared to the Ctrl group. # $p < 0.05$ and # $p < 0.05$ compared to the untreated SLE group.
